# Outcome of Metastatic Biliary Tract Cancer Harbouring *IDH1* or *FGFR2* Alterations: A Retrospective Observational Real-World Study from a French Cohort

**DOI:** 10.3390/jcm14196759

**Published:** 2025-09-24

**Authors:** Jean-Baptiste Barbe-Richaud, Fabien Moinard-Butot, Mathieu Cotton, Cécile Bigot, Pierre Rivière, Christine Belletier, Erwan Pencreach, Dan Karouby, Pascale Chiappa, Lauriane Eberst, Jean-Emmanuel Kurtz, Meher Ben Abdelghani

**Affiliations:** 1 Service D’oncologie Médicale, Institut de Cancérologie Strasbourg Europe (ICANS), 17 Rue Albert Calmette, 67200 Strasbourg, France; f.moinard-butot@icans.eu (F.M.-B.); c.bigot@icans.eu (C.B.); p.riviere@icans.eu (P.R.); c.belletier@icans.eu (C.B.); p.chiappa@icans.eu (P.C.); l.eberst@icans.eu (L.E.); je.kurtz@icans.eu (J.-E.K.); m.benabdelghani@icans.eu (M.B.A.); 2 Service de Pharmacie, Institut de Cancérologie de Strasbourg Europe (ICANS), 67200 Strasbourg, France; m.cotton@icans.eu (M.C.); d.karouby@icans.eu (D.K.); 3 Département de Génétique Moléculaire des Cancers, Hôpitaux Universitaires de Strasbourg (HUS), 1 Avenue Molière, 67200 Strasbourg, France; erwan.pencreach@chru-strasbourg.fr

**Keywords:** biliary tract cancer, *IDH1* mutation, *FGFR2* fusion, overall survival, real-world data

## Abstract

**Background**: Biliary tract cancer (BTC) management has undergone tremendous changes, benefiting from the identification of highly actionable molecular alterations. Among these, *IDH1* mutations and *FGFR2* fusions are the most common alterations detected and are classified as ESCAT tier 1 in BTC. However, their prognostic value in real-world settings remains uncertain. **Objective**: To explore overall survival (OS) in patients harbouring locally advanced or metastatic BTC (mBTC) with *IDH1* or *FGFR2* alterations, compared to those with wild-type tumours. **Methods**: This retrospective, multicentre study included patients with mBTC treated between 2020 and 2023 across five French centres. Patients were categorized into two cohorts based on molecular profiling: those with *IDH1* or *FGFR2* alterations, and those with wild-type tumours (WT-mBTC). **Results**: 119 consecutive patients were included. 18 were classified as altered (*IDH1* = 13; *FGFR2* = 5). Sixty-four pts underwent no molecular testing. The median OS of the entire cohort was 11.9 months (10.3–14.3). The median OS was 24.2 months (12.3–NA) versus 10.8 months (7.9–12.9), *p* = 0.02, in the altered and WT-mBTC cohorts, respectively. The Cox regression model conducted depicted an HR for death of 0.46 (CI95%, 0.2–0.9) for *IDH1* or *FGFR2* alterations. There were no diffence in PFS for first-line. **Conclusions**: Our cohort suggests that IDH1 or FGFR2 alterations may be associated with prognostic differences in patients with metastatic BTC, although they do not appear to influence outcomes under first-line treatment. These findings are consistent with trends observed in clinical trials. Whether improved survival is solely attributable to targeted therapies remains questionable. In line with ESMO recommendations, systematic molecular profiling should be considered in patients with mBTC.

## 1. Introduction

Biliary tract cancers (BTCs) are rare cancers (0.3–6 per 100,000 inhabitants per year) with both increasing incidence rates and geographical disparities [1]. According to the most recent TNM-AJCC-UICC classification [2], cases are divided into intrahepatic cholangiocarcinoma (iCCA, 10–20%), perihilar cholangiocarcinoma (pCCA, 50–60%), distal cholangiocarcinoma (dCCA, 10–20%), and gallbladder cancer. Distal and perihilar cholangiocarcinoma are often grouped into extrahepatic cholangiocarcinoma (eCCA). The strongest risk factors yet identified are lithiasis (hepatolithiasis, cholecystolithiasis and choledocholithiasis) and choledocal cyst [3], both associated with chronic inflammation and/or bile stasis which lead to carcinogenesis.

Most patients present with extensive disease at diagnosis, and a cure is an infeasible goal. The prognosis remains dismal, with an overall survival (OS) rate of less than 20% at 5 years [4].

For more than ten years, cisplatin-gemcitabine has remained the standard first-line therapy for metastatic disease, with a median OS of 11.7 months, a progression-free survival (PFS) of 8 months and an objective response rate (ORR) of 26.1% [5]. Recently, the TOPAZ trial demonstrated a significant improvement in OS with the addition of durvalumab, an immune-checkpoint inhibitor (ICI), to standard chemotherapy (CT), resulting in a median OS of 12.8 months, a PFS of 7.2 months and an ORR of 26.7% [6]. Few options remain after disease progression, with only the ABC-06 trial showing moderately improved OS with CT compared with the best supportive care [7].

The genomic characterization of BTC has revealed that the molecular landscape depends on the anatomical location of the tumour, with eCCA harbouring a similar molecular profile (mainly *TP53* or *KRAS* mutation) to that of pancreatic cancers [8]. However, a wider panel of molecular alterations, including mitochondrial gene alterations or chromatin regulators (i.e., *ARID1A*), is observed in iCCA [9]. Some of these alterations represent druggable targets that can be classified according to the ESMO Scale for Clinical Actionability of Molecular Targets (ESCAT), which ranges from I to V, with I indicating a strong and well-documented actionable target and V indicating a hypothetical target. Among iCCAs, mutations in *IDH1* and *BRAF*; fusions of *FGFR2*, *RET*, and *NTRK*; *HER2* overexpression; and MSI-H are classified as ESCAT I, with approximately one-third of iCCAs harbouring one of these genetic alterations [10]. Recently, the ClarIDHy trial showed promising results in pretreated mBTCs harbouring an *IDH1* mutation with ivosidenib, an *IDH1* inhibitor. In this trial, ivosidenib improved OS versus the placebo, with a median of 10.3 versus 5.1 months [11]. Likewise, futibatinib, an inhibitor of the *FGFR* pathway, demonstrated efficacy in a nonrandomized single group phase II trial among heavily pretreated patients, with a median OS of 21.7 months [12]. Both drugs received Food and Drug Administration (FDA) approval.

Herein, we present CLOUD, an observational study aiming to evaluate the real-life outcomes of *IDH1-* or *FGFR2-*altered mBTCs with those of WT mBTCs.

## 2. Materials and Methods

### 2.1. Patients

We performed a multicentre retrospective study between January 2017 and November 2023 in five French centres. Eligible patients were older than 18 years and were diagnosed with histologically confirmed, unresectable locally advanced or metastatic intrahepatic CCA, extrahepatic CCA or gallbladder cancer. Because no molecular screening was routinely performed before 2020, patients treated before 2020 were excluded, as were patients diagnosed with ampullomas. Patients were identified and recruited using institutional chemotherapy software.

Pretreatment patient characteristics, including demographic, clinical, and pathological data, as well as routine biology data, were collected. Molecular biology data were collected regarding the *IDH1* or *FGFR2* mutational status. All molecular analyses were performed with a next-generation sequencing (NGS) panel, using a commercial test (FoundationOne Medicine^®^ F. Hoffmann-La Roche, Basel, Switzerland) covering driver mutations, copy number variations and fusions [13]. Patients presenting with an *IDH1* or *FGFR2* alteration were grouped into the “altered” group, and those without such alterations or with no molecular screening performed were in the “wild-type” group. Radiological assessments were performed as clinically indicated by treating physicians according to the RECIST v1.1 criteria.

All patients received an information sheet and a non-opposition consent form for their registration within the database. All the data were obtained retrospectively, with no procedure taken to recover unavailable data by contacting healthcare providers or patients. This study was approved by the institution’s research committee (IRB 2024-05).

### 2.2. Objectives

The primary objective was to describe overall survival (OS) in the entire cohort, defined as the time from the diagnosis of de novo metastatic disease or metastatic relapse to death from any cause. The secondary objectives included the description of OS in patients with alterations, PFS in first-line therapy for the whole population, and PFS and disease control rate (DCR) in m-BTC patients treated with targeted therapy. PFS was defined as the time from the initiation of treatment with either chemotherapy or targeted therapy to the date of first radiological progression or death by any cause, whichever occurred first. DCR was defined as the percentage of patients who achieved a complete response (CR), partial response (PR) or stable disease (SD) with targeted therapy.

### 2.3. Statistical Analysis

Descriptive statistics were used to summarize patient demographics, clinical characteristics, and treatment patterns. Categorical variables are expressed as frequencies and percentages, and continuous variables are expressed as the means and standard deviations or medians and ranges. OS and PFS were estimated with the Kaplan-Meier method and are presented as medians and two-sided 95% CIs. The log rank test was used for statistical comparisons. A cox regression model was used after adjusting to potential confounders (adjustement on ECOG score, weight loss, age and histological differentiation). *p* values are two sided. All the statistical analyses were performed with R software (version 4.3.2).

## 3. Results

### 3.1. Patient Characteristics

**Figure 1:**
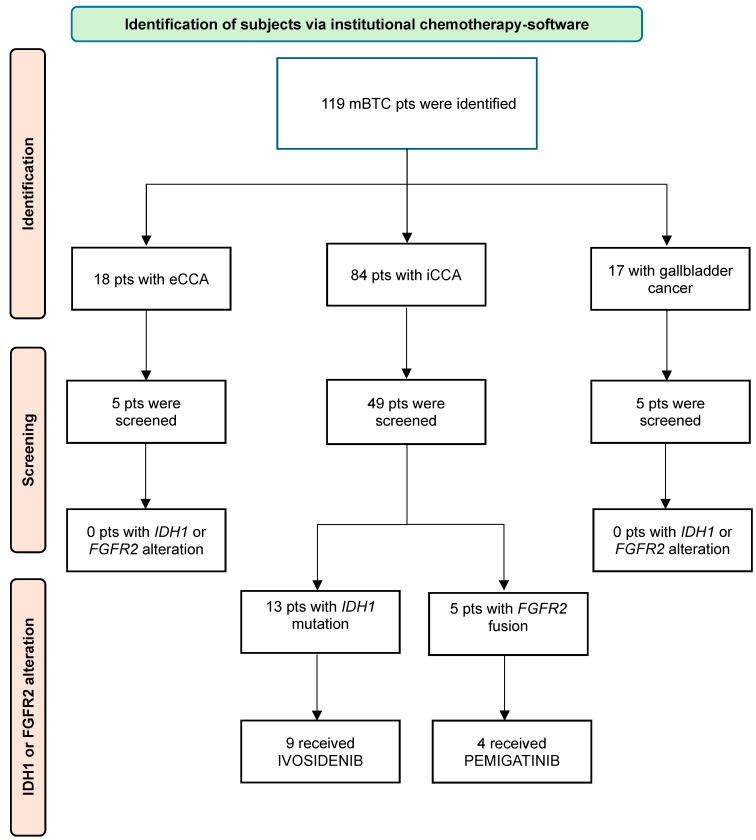
Flow chart. Pts: patients, mBTC: metastatic Biliary Tract Carcinoma, iCCA: intrahepatic Cholangiocarcinoma, eCCA: extrahepatic Cholangiocarcinoma.

From January 2020 through November 2023, 119 patients with metastatic or locally advanced BTC were included. The full details of patient inclusion are detailed in the flow chart (Figure 1).

The median age was 66 years (range: 31–85), and the majority of patients were female (56%). Most patients (67%) had an ECOG score of 0–1. Significant weight loss (>10%) was reported in 35% of cases. Histologically, 54% of tumors were well differentiated tumors. Disease was de novo metastatic in 69% of patients.

Eighty-four patients (71%) had iCCA. Eighty-two patients had metastatic disease at diagnosis (69%). The most common site of metastasis was the liver (61%). Ninety-nine patients (79%) received platinum-based therapy as the first-line treatment. Sixty-six patients (56%) received second-line treatment, and 34 patients (29%) received third-line treatment. The median time between diagnosis and molecular screening was 3.8 months. Most of the 64 patients who underwent molecular testing had iCCA (77%). Only eight patients had no mutations detected at all. The most common mutation reported was *TP53* (18 patients). A total of 13 patients (11%) had an *IDH1* mutation, and 5 patients (4%) had an *FGFR2* alteration. Apart from *IDH1* and *FGFR2* alterations, ESCAT I mutations were reported in 3 patients (two patients with *BRAF V600E* mutations and one patient with high MSI). The detailed demographic and clinical features of the patient cohorts are provided in Table 1.

Patients with mBTC harbouring either *IDH1* or *FGFR2* alterations (n = 18) were mostly female (67%), with a median age of 61 years. Among patients with *IDH1* mutations (n = 13), the *R132C* mutation was predominant (8 patients). Other mutations included *R172C*, *R132G*, *R132H*, and *G70S*. Comutations were reported in 6 patients. Nine patients received ivosidenib, the majority (5/9) of whom received third-line therapy. Six patients (6/9) received further therapy after progression with ivosidenib. Among patients with *FGFR2* alterations (n = 5), two presented with an *FGFR2-BICC1* fusion, one with an *FGFR2-CCDC141* fusion, one with an *FGFR-STK3* fusion and one with an *FGFR2* fusion with an unknown partner. Comutations were present in 4 patients. Four patients (4/5) were treated with pemigatinib, 3 patients (/4) as second-line treatment and one patient (/4) as third-line treatment. Table 2 summarizes the characteristics of patients harbouring an altered mBTC.

Patients with wild-type mBTC (n = 101) were mostly female (56%), with a median age of 67 years. Most patients had iCCA (65%). First-line therapy was a combination of cisplatin and gemcitabine for 68 patients (67%), and 13 patients (11%) received durvalumab in combination with CT. After progression, 41% of the patients received second-line chemotherapy, and 18% received third-line therapy.

### 3.2. Primary Objective

**Figure 2:**
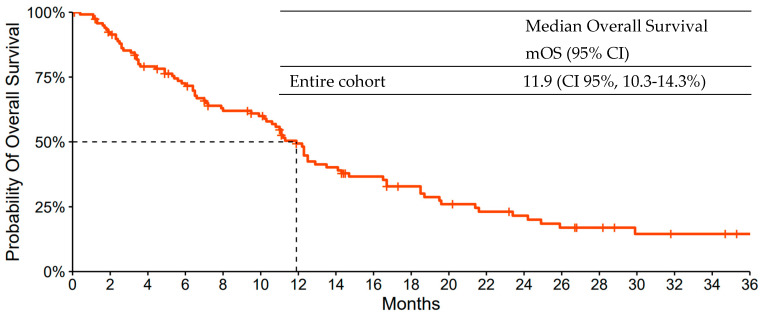
Kaplan-Meier Curve of Overall Survival of the entire cohort. CI denotes Confidence Intervals.

At the data cut-off, 95 patients (80%) had died. The median follow-up was 14.2 months. The median OS of the entire cohort was 11.9 months (95% CI, 10.3–14.3%) (Figure 2).

### 3.3. Secondary Objective

**Figure 3:**
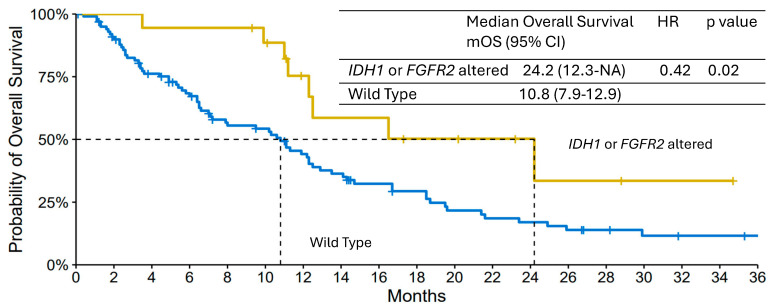
Kaplan-Meier Curves of Overall Survival of the *IDH1/FGFR2* altered group and wild type group. CI denotes Confidence Intervals.

The median OS was 24.2 months (95% CI, 12.3-NA) in the altered group and 10.8 months (95% CI, 7.9–12.9) in the WT group (*p* = 0.02) (Figure 3). The estimated 12-month OS was 75% and 44% in the altered and WT groups, respectively. The Cox regression conducted depicted an HR for death of 0.46 (95% CI, 0.2–0.9) for IDH1 or FGFR2 alterations (adjustement on ECOG score, weight loss, age and histological differentiation). A secondary analysis was conducted only on patients with iCCA (n = 84) since a fraction of iCCA patients in our cohort did not benefit from screening, and both *IDH1* and *FGFR2* alterations occurred preferentially in this topography. The median OS was 9.5 months (95% CI, 6.4–12.5) in the WT iCCA group (n = 66) compared with 24.2 months (95% CI, 12.3-NA) in the altered group (*p* = 0.009).

Finally, an analysis comparing only patients who benefited from molecular screening (n = 64) was conducted. The median OS was 13.5 months (95% CI, 11.1–23.4) for WT mBTC group (n = 46) compared with 24.2 months (95% CI, 12.3-NA) for altered mBTC group (*p* = 0.02).

The median PFS for patients treated with ivosidenib (n = 9) was 4.9 months, and the DCR was 44%. The median PFS for patients treated with pemigatinib (n = 4) was 4.1 months, and the DCR was 75%.

The median PFS during first-line therapy did not significantly differ between the two groups, with median PFS times of 7.9 months (95% CI, 5.6-NA) and 6.5 months (95% CI, 5.7–8.3) in the altered mBTC and WT mBTC groups, respectively (*p* = 0.9).

## 4. Discussion

Our study suggests that patients with IDH1 mutations or FGFR2 fusions may experience different clinical outcomes compared to those with wild-type mBTC. These findings support the importance of molecular testing in this population to identify potentially actionable alterations. The management of mBTC in patients with such molecular alterations could benefit from a tailored approach, including targeted therapies and appropriate supportive care. The management of mBTC has been revolutionized by the identification of targetable molecular alterations. The OS in the TOPAZ trial was approximately two times lower than the OS in FOENIX and almost the same as that in the ClarIDHy trial, which included pretreated patients [6,11,12]. These data suggest a plausible hypothesis of an alternative prognosis for patients presenting with mBTC harbouring *IDH1* or *FGFR2* alterations.

*IDH1* is highly expressed in the cytoplasm of liver cells and catalyse the oxidative decarboxylation of isocitrate to α-ketoglutarate (α-KG) while reducing NAD(P) to NAD(P)H [14]. Mutation of *IDH1* occurs mostly at the R132 position (arginine 132), leading to a reduction of α-ketoglutarate to 2-hydroxyglutarate (2HG), whose accumulation promotes biliary tract cancer by suppressing HNF4α (hepatocyte nuclear factor 4α), a key regulator of hepatocyte differentiation [15]. The prognostic value of *IDH1* mutations or *FGFR2* alterations in early-stage BTC has remained controversial [16]. At more advanced stages, the debate is also unresolved, with some data refuting a prognostic value [17,18], whereas others suggest better clinical outcomes for BTC harbouring *IDH1* alterations [19]. The prognostic value of *IDH1* in other tumour types is also controversial. *IDH1* mutations have been identified in most gliomas and in 10% of glioblastomas and are associated with favourable outcomes [20]. Their prognostic value is still unknown in chondrosarcoma patients [21]. Nevertheless, the development of *IDH* inhibitor therapy improved OS in patients with BTC [11] and acute myeloid leukaemia [22] and dramatically improved PFS in patients with low-grade glioma [23].

The FGF pathway consists of four transmembrane receptor tyrosine kinases, FGFR1–4, and is deeply involved in differentiation, proliferation and essential cell mechanisms. Consequently, dysregulating the FGF signalling network may lead to tumorigenesis [24]. BTC with FGFR2 alterations is likely to have a unique pattern of evolution. Early-stage BTC harbouring *FGFR2* alterations seems to have better OS and disease-free survival (DFS) than BTC with wild-type *FGFR* [25,26]. Similar conclusions have been drawn at more advanced stages. Jain et al. reported a median OS of 37 months at the metastatic stage for BTC harbouring *FGFR2* alterations versus 20 months for WT-BTC [27]. However, the difference in OS was not associated with a better response to first-line therapy in the published data [28]. Whether the difference in OS at more advanced stages is linked to targeted therapy is still uncertain [28]. Jain et al. reported a cohort with a median OS of 24.3 months for BTC patients with *FGFR2* alterations after excluding patients treated with targeted therapy versus 20 months for wild-type tumors [27] in favour of the natural course of a specific disease regardless of targeted therapy.

Analytic assessment of molecular alterations remains crucial. In our study, all the molecular tests performed used an NGS method on the tumour biopsy. Recent studies have demonstrated that droplet digital PCR (ddPCR) could be an alternative to NGS for identifying *IDH1* variants while reducing the cost and the technical time with high specificity and sensitivity [27]. The standard technique used in clinical trials to detect *FGFR2* is still NGS [29]. Zou et al. recently investigated immunochemistry (IHC), with poor results, whereas fluorescence in situ hybridization (FISH) achieved results that were more consistent with those of NGS [30]. FISH could soon become a standard technique because of the feasibility of performing NGS testing in cases of ambiguous FISH, such as in the diagnosis of anaplastic lymphoma kinase (ALK) fusion in non-small cell lung carcinoma (NSCLC) [31]. Another approach is RNA-based diagnosis, which can detect gene fusions with high sensitivity and sensitivity but presents several challenges [32], one of which is the lack of remaining tumour material after standard diagnostic analysis.

Liquid biopsy is routinely performed in the management of NSCLC, but it is not well established in BTC. A recent study by Berchuck et al. demonstrated high concordance between NGS performed on liquid biopsy and NGS on tissue biopsy for the detection of *IDH1* mutations (87%), but poor results were reported for *FGFR2* fusions (18%) [33], suggesting that *IDH1* mutations can be detected if no tissue is available. The detection of *FGFR2* alterations in clinical trials is based on NGS of tissue samples. However, exploratory analysis from the FOENIX trial [12] revealed 87% positive percent agreement (PPA) between assessments of tissue and ctDNA. These contradictory data highlight the need for additional studies to clarify these results.

However, the techniques employed to detect specific molecular alterations in BTC are hampered by the landscape of molecular alterations, which may prevent the identification of targetable mutations. Moreover, a complete molecular landscape could lead to increased depth in personalized medicine, considering the effects of comutations on targeted therapies, as has already been demonstrated in NSCLC with *TP53* [34]. Notably, NGS based on DNA can still face challenges in detecting gene fusions because of the possibility that the breaking point occurs in a large intron that cannot be covered by the DNA panel, which is focused on exons [35].

The promising results of targeted therapies in advanced lines of treatment have led to their evaluation in BTC as first-line maintenance therapy after 4 cycles of immunochemotherapy (NCT05615818). New clinical trials are also evaluating the efficacy of targeted therapy for molecular alterations identified in advanced or metastatic cancer (NCT04116541).

A remaining challenge is access to molecular screening as well as to targeted therapy and its management [36]. Molecular assessment requires a specialized laboratory, usually available only in university hospitals, highlighting the necessity of cooperation between nonuniversity hospitals and referral centres. Despite the poor prognosis of these rare tumours, BTC management remains challenging, and patients should be enrolled in clinical trials when feasible, underscoring the need for cooperation between hospitals.

Linked to its poor prognosis, early identification and prevention represent another key axis in the management of BTC. Lithiasis of the biliary ducts is a strong risk factor associated with BTC. However, biliary lithiasis is one of the most common conditions in gastroenterology, which highlights the need to identify patients who may benefit from active monitoring with regular follow-up, and those who may require specific interventions [37]. Current data suggest that surgical or combined treatments in selected cases of hepatolithiasis—defined as biliary stones located above the hilar confluence—or choledochal cysts, significantly reduce the risk of developing BTC [38,39,40]. Additionally, radiological assessment can be challenging for the identification of iCCA, underscoring the importance of collaboration between secondary and tertiary centers when iCCA is suspected [41].

Our study results support the possibility of better outcomes in patients harbouring *FGFR2* alterations or *IDH1* mutations but has several limitations. First, this was a retrospective descriptive study with a limited sample size which may affect the accuracy and completeness of the data and limits the strength of any conclusions. Moreover, owing to the small number of patients harbouring *IDH1* mutations or *FGFR2* fusions, these patients are combined into one unique subgroup, which does not allow us to evaluate the separate outcomes for *IDH1* mutants and *FGFR2* alterations. Finally, some of the patients in the WT group did not undergo molecular screening, particularly those with eCCA or gallbladder cancer introducing a potential selection bias. Targetable alterations are less frequently observed in eCCA than in iCCA, leading to physicians choosing not to screen them. Notably, the median OS observed in the nonmutated group and in the overall cohort corresponded to the OS of the ABC02 trial evaluating cisplatin–gemcitabine in first-line mBTC treatment, indicating consistency with meaningful real-life data [5].

## 5. Conclusions

Our real-world French cohort is consistent with clinical trial data, suggesting that *IDH1* or *FGFR2* alterations in biliary tract cancer have a prognostic impact. Molecular testing allows the identification of highly actionable alterations and offers patients access to personalized treatment after the failure of first-line chemo-immunotherapy. Given the improved outcomes associated with targeted therapies, ESMO recommends systematic molecular testing for all patients.

## Figures and Tables

**Table 1:** Patient’s Characteristics.

Characteristics	Entire Cohort N = 119 (%)
Median age (range)—years	66 (31–85)
Sexe—no. (%)	
Male	51 (44)
Female	68 (56)
ECOG performance status score—no. (%)	
0–1	80 (67)
2 or more	39 (33)
Weight loss > 10%	41 (35)
Primary Tumor Type—no. (%)	
Intrahepatic cholangiocarcinoma	84 (71)
Extrahepatic cholangiocarcinoma	18 (15)
Gallbladder	17 (14)
Histological differentiation—no. (%)	
Well differentiated	64 (54)
Poorly differentiated	53 (45)
Unknown	2 (2)
Disease Status	
Initially Unresectable	82 (69)
Recurrent	37 (31)
Tumor > 5 cm—no. (%)	45 (38%)
Biliary stent—no. (%)	22 (19%)
Metastatic site—no. (%)	
Liver	72 (61)
Lymph node	52 (44)
Pulmonary	34 (29)
Molecular Screening—no. (%)	
Yes	64 (54)
Intrahepatic cholangiocarcinoma	49 (77)
Extrahepatic cholangiocarcinoma	10 (16)
Gallbladder	5 (8)
No—Unknown	55(46)
Molecular Alteration—no. (%)	
IDH1	13 (11)
FGFR2	5 (4)
First Line Therapy—no. (%)	
Cisplatin-Gemcitabine-Durvalumab	13 (11%)
Gemcitabine-Platinum	81 (68%)
Gemcitabine	25 (21%)

**Table 2:** Patients Baseline Characteristics of the IDH1/FGFR2 altered group.

Characteristics	No. of Patients N = 18 (%)
Median age (range)—years	61 (46–80)
Sexe	
Male—no. (%)	6 (33)
Female—no. (%)	12 (67)
Primary Tumor Type no. (%)	
Intrahepatic	18 (100)
Disease Status—no. (%)	
Initially Unresectable	13 (72)
Recurrent	5 (28)
Mutation	
*IDH1* Mutation	13 (72)
* IDH1 R132C*	8 (62)
Co-mutation	6 (46)
* FGFR2* alteration	5 (28)
* FGFR2-BICC1*	2 (40)
Co-mutation	4 (80)
First-Line Of Therapy—no. (%)	
Cisplatin-Gemcitabine-Durvalumab	3 (17)
Platinum-Gemcitabine	13 (72)
Gemcitabine	2 (11)
Patients receiving IVOSIDENIB—no. (%)	9 (69)
Second-Line	3 (33)
Third-Line	5 (56)
Fourth-Line	1 (11)
Patients receiving treatment after progression	6 (67)
Patients receiving PEMIGATINIB—no. (%)	4 (80)
Second-Line	3 (75)
Third-Line	1 (25)
Patients receiving treatment after progression	1 (25)

## Data Availability

The original contributions presented in this study are included in the article. Further inquiries can be directed to the corresponding author.
